# RACIAL DIFFERENCES IN CORRESPONDENCE BETWEEN OBJECTIVE HEARING AND SUBJECTIVE HEARING RATINGS

**DOI:** 10.1093/geroni/igad104.3083

**Published:** 2023-12-21

**Authors:** Charity Lewis, Joanne Elayoubi, Michelle Arnold, Yuri Jang, Victora Sanchez, Julia Toman, William Haley

**Affiliations:** University of South Florida, Tampa, Florida, United States; University of South Florida, Tampa, Florida, United States; University of South Florida, Tampa, Florida, United States; University of Southern California, Los Angeles, California, United States; University of South Florida, Tampa, Florida, United States; University of South Florida, Tampa, Florida, United States; University of South Florida, Tampa, Florida, United States

## Abstract

**Background:**

Hearing loss and hearing health are underrecognized public health urgencies affecting over 70% of adults aged 70 and older. Untreated hearing loss has its association with higher prevalence of adverse physical and cognitive health outcomes. Current literature reveals racial disparities in hearing aid utilization. Based on the Health Belief Model suggesting that subjective beliefs are predictive of health behaviors, the present investigation focused on the correspondence between objective and subjective ratings of hearing in older Blacks and Whites. We identified groups that are concordant and discordant (overestimation and underestimation) in their ratings and explored determinants of the group membership.

**Methods:**

We included participants over age 50 from the Health and Retirement Study (N = 2,495). Subjective and Objective hearing loss relation to race and age was determined via Chi-square analysis. Hierarchical linear regression models were completed to identify odds ratios for overestimation and underestimation of hearing.

**Results:**

Whites aged 50-64 and 65-75 were significantly more concordant in their ratings than Blacks. Blacks aged 65-75 had significantly higher rates of overestimation of hearing. In a fully adjusted model for overestimation of hearing, Blacks had significantly greater odds of overestimation of hearing than Whites (OR = 1.47), and overestimation was significantly greater with higher age (OR = 1.03) and with tobacco use (OR = 1.29).

**Conclusion:**

While mechanisms explaining lower hearing aid utilization in Black older adults need further study, perceived severity (consistent with the Health Belief Model) may be one component of reduced hearing aid use in Black older adults.

